# The cytoskeletal arrangements necessary to neurogenesis

**DOI:** 10.18632/oncotarget.6838

**Published:** 2016-01-07

**Authors:** Claudia Compagnucci, Fiorella Piemonte, Antonella Sferra, Emanuela Piermarini, Enrico Bertini

**Affiliations:** ^1^ Department of Neuroscience, Unit of Neuromuscular and Neurodegenerative Diseases, Children's Research Hospital Bambino Gesù, Rome, Italy

**Keywords:** neurogenesis, cytoskeleton, induced pluripotent stem cells (iPSCs), actin, tubulins

## Abstract

During the process of neurogenesis, the stem cell committed to the neuronal cell fate starts a series of molecular and morphological changes. The understanding of the physio-pathology of mechanisms controlling the molecular and morphological changes occurring during neuronal differentiation is fundamental to the development of effective therapies for many neurologic diseases. Unfortunately, our knowledge of the biological events occurring in the cell during neuronal differentiation is still poor. In this study, we focus preliminarily on the relevance of the cytoskeletal rearrangements, which earlier drive the morphology of the neuronal precursors, and later the migrating/mature neurons. In fact, neuritogenesis, neurite branching, outgrowth and retraction are seminal to the development of a fully functional nervous system. With this in mind, we highlight the importance of iPSC technology to study the processes of cytoskeletal-driven morphological changes during neuronal differentiation.

## INTRODUCTION

The beginning of *in vivo* neurogenesis requires the commitment of the embryonic stem cells (ESCs) to the epithelial fate, which converts a round-shaped ESC into a bipolar cell with recognizable apico-basal and medio-lateral axes [1]. The following phase of differentiation consists in the development of neuronal precursors, with a characteristic bipolar and elongated morphology. Following this cytoskeletal reorganization, the neuronal progenitors undergo a series of finely controlled events, such as the formation and development of neurites (neuritogenesis) and the subsequent maturation of one neurite into an axon (axonogenesis) and of the other neurites into dendrites (dendritogenesis) as well.

**Figure 1:**
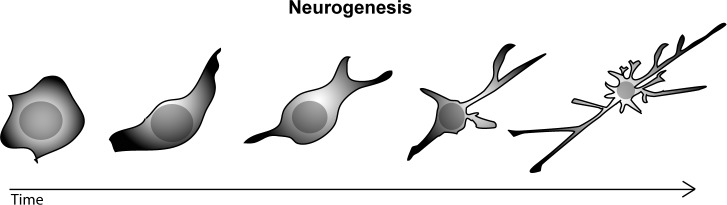
Drawing of a stem cell differentiating into a mature neuron, with well developed axon and dendrites

Following neuronal commitment, rounded neuronal precursors make membrane sprouts, which later develop into neurites and are extended as the neurons differentiate (neurite outgrowth) (Figure 1). Extending neurites generate branches (neurite branching), leading to axon collaterals or dendritic arbors, or they exhibit transient retraction (neurite retraction). Cytoskeletal components not only control cell morphology, but they also form a scaffold for organelle (i.e., mitochondria) transport (i.e. microtubules or MTs) and they regulate growth cone motility and axon guidance (i.e. actin filaments or AF).

The cytoskeleton of eukaryotes is composed of filamentous proteins belonging to three major families of elements: 1) the microtubules or MTs (25 nm of diameter, made by dimers of α and β-tubulin), 2) actin filaments or AF (6 nm of diameter) and 3) intermediate filaments or IF (10 nm of diameter). Recent evidence show that IFs (homopolymers or heteropolymers) have direct and indirect roles in cytoskeletal rearrangements, cell adhesion, cell mechanical properties and intracellular signaling [2].

## NEURITOGENESIS AND THE CYTOSKELETON

Neuronal precursors at first give rise to membrane sprouts consisting of AF-rich lamellipodia [3, 4]. The membrane sprouts subsequently mature to become short neurites containing a core of MTs. These processes require rearrangements of the cytoskeleton in all of its components, and these mechanisms need to be functionally coordinated and integrated. For example, cell movements are dependent on the dynamic equilibrium between the globular monomeric state and the polymeric filament state of Actin (G-actin and F-actin, respectively) [5]. The formation of MTs is initiated by the binding of αβ-tubulin heterodimers to the γ-tubulin ring complex on the surface of an MT organizing centre such as the centrosome [6,7]. MT elongation occurs through addition of tubulin heterodimers to the plus end, thus forming a polarized cytoskeleton. In fact, MTs undergo cycles of growth and shortening in a process called ‘dynamic instability’ [8, 9]. Moreover, actin filaments grow steadily, while MTs undergo dynamic instability, which allows the microtubule cytoskeleton to be remodeled rapidly [10]. Intermediate filaments (IF) are cell-type specific non-polar cytoskeletal filaments (i.e. keratins are found in trichocytes and epithelial cells, desmin in myogenic cells, nestin in the neuronal progenitors and lamins in the cell nucleus). The family of the neural IF includes glial fibrillary acidic proteins (found in glial cells), vimentin (in cells of mesenchymal origins), synemin (in astrocytes), the neurofilament-light (NF-L), neurofilaments-medium (NF-M), and neurofilament-heavy (NF-H) chains (in central and peripheral nervous system), α-internexin (in central neurons), and nestin (in neuroepithelial cells) [11-13].

## CYTOSKELETAL REARRANGEMENTS AND THE EXTRACELLULAR ENVIRONMENT

Neuritogenesis requires a profound cytoskeletal reorganization, and among the factors controlling these events is the interaction with the extracellular environment and, in particular, with several extracellular matrix ligands. To this aim, the role of proteins communicating between the extracellular and intracellular environment is fundamental. For example, the proteoglycan NG2 (or neural/glial antigen 2) is able to mediate interactions with both the extracellular matrix and the actin cytoskeleton, thus initiating an active signalling between extracellular and intracellular environments [14]. Interestingly, there is the general agreement that NG2 cells represent an immature neural cell population that, under differing environmental conditions, can terminally differentiate into mature neural cell types [15]. NG2 is a transmembrane protein, and its extracellular domain includes sites characterized by disulfide bonds, chondroitin sulfate chain and domains which are readily cleaved by a variety of proteases [16]. The biological relevance of this cleavage is unclear: one possibility is that it is a mechanism to release NG2 from a putative receptor *via* regulated proteolysis. The intracellular domain includes threonine phosphorylation sites, PKC target, whose phosphorylation state regulates cell behaviour such as spreading and migration [17-20]. The interest of NG2 cells is linked to its ability to mediate extracellular signaling to the intra-cellular environment in physiologic condition, and importantly, NG2 cells also react to injuries or pathological conditions with morphological changes, increased proliferation rate and activation of migratory process that leads to accumulation of NG2 cells in the lesioned area. NG2 cells respond also to progressive neurodegenerative insults, including Alzheimer's disease (AD) and Amyotrophic lateral sclerosis (ALS). Several questions remain to be answered concerning the real role of NG2 in the central nervous system, in particular the questions related to the mechanism of modulation of the neuronal network and the response to pathological conditions.

## NEURITOGENESIS AND THE BREAK OF SYMMETRY

One of the key and initial events occurring during neurogenesis is the break of the initial symmetry. In fact, a neuronal progenitor encounters three levels of symmetry breaking before giving rise to a neuron, *i*) the first being the choice between symmetric and asymmetric cell division, *ii*) the second being the first neurite sprouting and *iii*) the third being the asymmetry present in the molecular organization of the MTs, which is necessary to the mature neuron to function properly. The primary progenitor cells of the central nervous system are the neuroepithelial cells, which characteristically exhibit apical-basal polarity [21]. A key feature of proliferative division of the neuroepithelial cells and of the radial glial cells is that cleavage occurs along their apical-basal axis [21]. In fact, the appearance of the morphological break of symmetry, leading to the appearance of the first neurite, has its molecular basis in the break of molecular symmetry. If one progenitor cell gives rise to two identical daughter cells (containing qualitatively and quantitatively the same cellular content), the division is considered symmetric because the plane of division is perpendicular to the lumen of the neural tube, and the daughter cells will be two progenitor cells. On the contrary, if the plane of division is not perpendicular to the neural tube lumen, the apical plasma membrane of the neuroepithelial cells will be bypassed (rather than bisected) by the cleavage furrow and therefore, it will be inherited only by one of the daughter cells. The two daughter cells will inherit different cellular contents and the resulting cells will give rise to one progenitor cell and to one neuronal precursor [22]. In particular, the biological mechanisms controlling the break of symmetry during neuronal development in mammals have been investigated by Fish et al. [22]. They focused on the protein ‘Abnormal Spindle-like, Microcephaly-associated’ (or ASPM), which by *in situ* hybridization studies on murine embryos was shown to be expressed around the onset of neurogenesis in the proliferative ventricular zone of the forebrain [22]. In their studies, the knockdown of *Aspm* in mice (by *in utero* electroporation of short interfering RNAs) has severe effects on centrosome localization in the mitotic phase of neuroepithelial cells and perturbs vertical cleavage plane orientation, leading to asymmetric cell division and to an increased neuron-like fate of the neuroepithelial cell progeny [22]. Interestingly, the Drosophila homologue of ASPM, Asp, also has a crucial role at spindles pole during mitosis. In particular, Asp may have a role in focusing microtubules, including those of the central spindle, a structure relevant for the positioning of the cleavage furrow [23, 24]. Moreover, consistent with a role of ASPM in regulating the size of the neocortex (which derives from the embryonic forebrain), the primate and human lineages present strong positive selection for evolutionary changes in the Aspm protein [25, 26].

**Figure 2:**
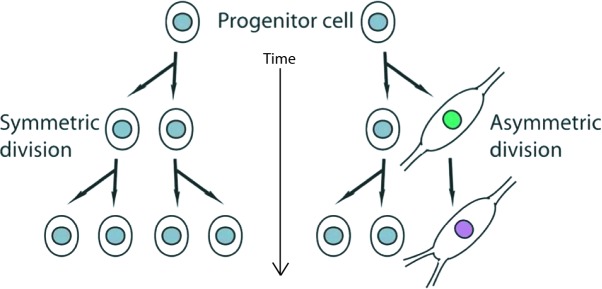
Schemata depicting the organization of proliferating stem cells (progenitor cells in this context) undergoing symmetric cell divisions (on the left side of the panel) The right side of the panel depicts a progenitor cell ongoing asymmetric division, where one daughter cell remains proliferative (indicated by the grey nucleus), while the other (on the right) is committed toward neuronal differentiation (indicated by the green nucleus, and becoming even more committed with time, as indicated by the lavender nucleus).

In conclusion, ASPM, which is located at mitotic spindle poles of neuroepithelial cells, have been found to control the maintenance of the cleavage plane orientation, thus regulating the switch between symmetric, proliferative divisions of neuronal progenitors *versus* the asymmetric divisions giving rise to one neuronal progenitor and one differentiating neurons during brain development [27] (Figure 2). Importantly, mutations in ASPM are responsible for a form of primary microcephaly observed in humans.

**Figure 3:**
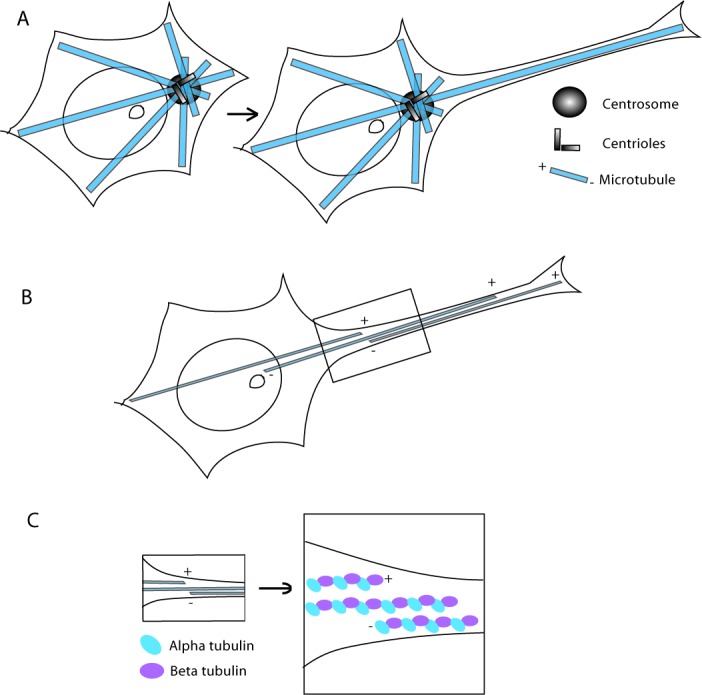
Figure depicting the organization of the microtubules in a stem cell and in a developing neuron **A**. Drawing depicting the changes of the MT network during neuritogenesis. **B**. Drawing of a neuron where it is clear that the MTs are oriented with minus ends towards the cell body and plus ends away from the cell body. In **C.** a close up view of the box in B is reported, where α- and β- tubulins have been highlighted (in light blue and lavender, respectively).

Another level of symmetry breaking during neurogenesis consists in defining the position of the centrosome, which after cell division matches with the site of axonal sprouting. Correlations of centrosome localization to the axon formation site have been observed during the early phase of axonogenesis in several types of neurons [28-30]. In fact, shortly after plating hippocampal neurons on a substrate in culture, centrosomes, together with the Golgi apparatus and clusters of endosomes, accumulate beneath the first neurite that later develops into an axon (Figure 3A). Furthermore, neurons with multiple centrosomes develop multiple axons. Whether the location of the centrosome is cause or consequence of axonal positioning is still under debate. What is the exact mechanism by which an instructive role is given to one pole of the neuronal precursor to determine axon orientation is still a matter of debate. In the past, the centrosomal localization has been proposed to be the leading mechanism, but currently, Dotti's group suggests that it is the localization of N-cadherin that specifies the first asymmetry in developing neurons [31]. At first several neurites develop, among these one neurite further develop as an axon and the others as dendrites, leading to the establishment of functional neuronal polarity [3]. The determination of axon fate depends on the highly coordinated and integrated activity of MTs and AFs in neurites. Recently, MT stability has also been demonstrated to be a signal specifying neuronal polarization [32].

Another level of asymmetry is found at the molecular level in MTs, in fact α- β tubulin dimers differentiate the two ends of the microtubule: the minus end begins with α -tubulin, and β -tubulin is exposed at the growing plus end. Microtubules continuously switch between plus end growth and shrinkage in the process of dynamic instability [33]. In proliferating cells, most of the nucleation sites are present at the centrosome, so microtubule minus ends are located near the center of the cell and plus ends at the periphery, leading to a radial organization [34, 35]. On the contrary, in most differentiated animal cell types (i.e. muscle, epithelial and neuronal cells, as well as most fungi and vascular plant cells), MTs are arranged in a non-radial pattern [34]. Further studies show that axons and dendrites have distinct arrays of MTs. In axons, MTs are usually long and oriented uniformly, their plus ends distal to the cell body, whereas in dendrites MTs are shorter and they exhibit a mixed polarity [35]. In particular, a radial MT organization would not be possible in neurons, where single microtubules (that could be 100 μm long) would need to extend from the centrosome to the cell body through the entire length of the axon or dendrite (which might need to be much longer than 100 μm) [36]. In neurons, MTs form overlapping arrays in axons and dendrites [37], thus, the minus and plus ends are scattered throughout axons and dendrites. Despite this may appear a random organization, it is not, in fact, in vertebrate axons (but not in dendrites) all MTs are oriented with minus ends towards the cell body and plus ends away from the cell body (Figure 3B, 3C) [37, 38].

Interestingly, the asymmetry linked to centrosome localization is relevant to control both the asymmetric cell division taking place in neural precursors and in establishing the molecular microtubular asymmetric organization. In fact, the knockdown of Aspm (the protein responsible for regulating asymmetric *versus* symmetric cell division) in murine models shows alterations of the centrosome localization [22], the initial axon forms close to the position where the centrosome reside [39]. In addition to this, it is important to understand that despite these biological events may seem independent one to the other, they are indeed strictly integrated as centrosomes, the Golgi apparatus and endosomes cluster together close to the area where the first neurite will form, which is in turn opposite from the plane of the last mitotic division [39].

In addition to these studies, a role for the Golgi apparatus in controlling axoplasmic flow is emerging. In particular, Bradke et al. [40] demonstrated that axonogenesis is preceded by an increased amount and greater transport of membrane organelles, a higher concentration of mitochondria and peroxisomes, ribosomes and of cytosolic protein. The authors suggest that among the determinants of neuronal morphological polarization, a well organized cytoplasmic flow is necessary and, that functional polarity is established by later molecular sorting events [40]. In this context, the Golgi apparatus (where newly synthesized proteins are segregated) has a strong impact in directing the transport of the newly synthesized proteins to the axonal or dendritic surface [41]. Another important role for the Golgi apparatus in neurogenesis is its role in non-centrosomal microtubule nucleation. In fact, Stiess at al. [42] showed that centrosome loses its function in microtubule organizing center in rodent hippocampal neurons. Following this, Ori-McKenney et al. [43] demonstrated that non-centrosomal microtubule nucleation in neurons is organized by Golgi outpost. Recently, Yalgin et al. [44], performed a screening for branching-control effectors in Drosophila sensory neurons and identified Centrosomin, a centrosome-associated protein for mitotic spindle maturation. In particular, Centrosomin is localized to the Golgi cis face and it recruites microtubule nucleation to Golgi outposts. Interestingly, removal of Centrosomin caused increased branching, thus suggesting that the location of Centrosomin to Golgi outposts is important to guide microtubule polymerization [44].

In addition to the role of the cytoskeleton in controlling cellular morphology, regulating and maintaining neuronal polarization, it also has an active function in the transport of proteins, vesicles and organelles along the axon and the dendrites. In particular, three classes of motor proteins, transporting the cargoes along the cytoskeleton, exist: myosins (moving along actin filaments), kinesins and dyneins (moving along the microtubules) [45]. Kinesins are responsible for the anterograde transport of axonal proteins (as it moves towards the plus end), while dynein controls retrograde transport (moving towards the minus end) [46, 47]. Since the protein synthesis mainly takes place in the cell body (which can be only 0.1% of the total cell volume), the growth and maintenance of neuronal processes requires a finely controlled delivery of materials to axons and dendrites [48]. Therefore, the materials necessary to accomplish the neuronal functions have to be supplied by mechanisms involving transport. [49]. For these reasons, it is not surprising that an increasing amount of neurodegenerative disorders is linked to abnormalities in transport-related proteins [47, 50]. For example, mutations in the gene *KIF5A* are linked to Hereditary Splastic Paraplegia (HSP) and Charot-Marie-Tooth disease type 2 (CMT2) [51].

An additional process that occurs during neurogenesis is the axonal transport of messenger RNAs (mRNAs). In fact, post-transcriptional regulation of mRNA trafficking and metabolism is crucial in both differentiating and differentiated tissues. In particular, in neurons, where long distances occur between the cell body, where the mRNA is synthesized, and the synapse of the axon, where the product of mRNA translation (the protein) is required, the subcellular pre-localization of mRNAs and the locally regulated translation has several advantages: i) a high number of proteins can be obtained locally by transporting and translating a single mRNA molecule; ii) some proteins may be harmful to the cells when synthesized in the incorrect location; iii) in response to local signaling, proteins may be synthesized only in compartment exposed to the signal in order to regulate a differential translation. [52]. The axonal and localized protein synthesis gives neuronal processes the possibility to respond rapidly to extracellular stimuli. Moreover, locally synthesized axonal proteins enable neurons to direct their growth and to respond to guidance cues, together with helping to initiate regeneration following injury. Ultrastructural studies suggest that axons do not have rough endoplasmic reticulum or Golgi apparatus, but growing axons with protein synthetic activity contain endoplasmic reticulum (or ER) and Golgi components needed for classical protein synthesis and secretion [53]. In conclusion, recent RNA profiling studies on axons of cultured neurons have shown that hundreds of different mRNAs are known to be transported into axons [54-56].

## THE DYNAMIC INSTABILITY OF MICROTUBULES IN DEVELOPING NEURONS

Before the establishment of neuronal polarity or during neuritogenesis, the future axon contains more stable (acetylated) MTs *versus* dynamic (tyrosinated) MTs than other neurites. After the establishment of polarity, the axon maintains higher levels of stable MTs. In support of this, it has been observed that low doses of the MT destabilizer ‘nocodazole’ impair dendrite formation without affecting axonal development. Despite the increasing number of signaling molecules regulating the dynamics of MTs and AFs that affect neuronal polarity [57], the molecular determinants are not fully understood. Therefore, further studies are needed to understand the bases of microtubule dynamic instability in developing and mature neurons.

To expand on the cytoskeletal features of developing neurons, it is known that in migrating neurons, the MTs and AFs extensively overlap in the leading process, while in mature neurons, MTs are longitudinally oriented at the core of the neurite and they end in the central domain of the growth cone, actin filaments, on the opposite, are distributed throughout the growth cone [58, 59]. The understanding that a fine regulation of the cytoskeletal components is necessary for the proper development of neurons, suggest that the processes of neuritogenesis and development of the mature neurons are strictly controlled at the molecular and biochemical levels.

## OXIDATIVE CONTROL OF THE CYTOSKELETON

Several studies suggest that alterations of the MT dynamics in susceptible neurons would explain the dying-back phenomena observed in neurodegenerative disorders (i.e. Alzheimer's disease, Parkinson's disease, Amyotrophic Lateral Sclerosis and Friedreich's ataxia) [60, 61] and since MT alterations can be mediated by oxidative damage, it is relevant to understand the meachanisms of oxidative control of the cytoskeleton. A mechanism mostly understudied is the control that oxidative changes of the intracellular environment exerts on the cytoskeleton. Reactive oxygen species (ROS) [primarily superoxide [O_2_-·] and hydrogen peroxide (H_2_O_2_)] contribute to the generation of other radicals or oxidative species, increasing the cellular state of oxidation and for this reason they need to be detoxified intracellularly. Glutathione is considered an antioxidant that protects the cells against oxidative stress. Glutathione (GSH) is a tripeptide thiol antioxidant molecule, present within cells at millimolar concentrations (1-10mM). In response to increased oxidation, glutathione becomes oxidized to its disulfide form (GSSG). The disulfide form, GSSG can be reduced back to GSH *via* glutathione reductase, at the expense of oxidation of NADPH. In the cytosol, the ratio of oxidized to reduced GSH is around 100:1, while in the ER, which has a more oxidizing environment to permit oxidative protein folding, the ratio of GSH/GSSG is around 3:1 [62]. The extent of oxidation of GSH is often used as a measure of “oxidative stress” (which can be measured in various biological fluids, including plasma). Therefore, the loss of reduced glutathione and formation of glutathione disulfide is considered a parameter of oxidative stress, which is increased in several diseases (i.e. myocardial contraction, hypertrophy and inflammation) [63]. Importantly, glutathione contributes to the degradation of H_2_O_2_ through glutathione peroxidase (GPx), generating GSSG. Finally, the oxidized glutathione is recycled by glutathione reductase (GR). This mechanism is activated when H_2_O_2_ detoxification by catalase is overloaded (for example in pathological conditions), or when this pathway is absent, as in mitochondria [64]. When GSSG recycling is over-loaded, the concentration of GSSG becomes high enough to induce the formation of glutathione-protein adducts (PS-SG). This modification is able to alter the function of proteins, inactivating or activating them, depending on the protein [65].

The event of protein glutathionylation depends on several mechanisms including: 1) GSH levels (as GSH depletion can decrease glutathionylation); 2) GSH/GSSG ratio (an oxidizing ratio will increase protein glutathionylation *via* thiol disulfide exchange reactions); 3) Glutaredoxins (in fact, with a high GSH/GSSG ratio, Grx will act as a deglutathionylating enzyme, while in oxidizing conditions it can catalyse glutathionylation).

Post-translational *S*-glutathionylation occurs through the reversible addition of a proximal donor of glutathione to thiolate anions of cysteines in target proteins, where the modification alters molecular mass, charge, and structure/function and/or prevents degradation from sulfhydryl overoxidation or proteolysis. Importantly, catalysis of the forward (glutathione *S*-transferase P) and reverse (glutaredoxin) reactions creates a mechanism that can also control certain protein functions (activation, inactivation, loss of function, and gain of function), including those involved in cytoskeletal arrangements [66, 67]. In fact, it has been reported that several proteins of the cytoskeleton can be glutathionylated (i.e. actin), and this can easily affect the cytoskeletal stability and cellular functionality [68]. Thus, a deeper understanding of the control that the oxidative state exerts on the cytoskeleton is essential to pave to way to future clinical applications, able to prevent and/or treat neurologic diseases caused by cytoskeletal alterations.

## CYTOSKELETAL DISRUPTIONS AND NEUROLOGIC DISEASES

Genetic investigation in humans and mice have revealed that several mutations occur in genes involved in biological processes controlling the development of a functional nervous system (i.e. cell proliferation, differentiation, migration, adhesion, cytoskeletal dynamics), and they lead to altered neurologic development. Earlier is the insult during neurogenesis, more severe the neuronal phenotype is. Since the cytoskeleton is involved in a broad series of events regulating neurogenesis and maintenance of the neuronal function, it is easy to understand that alterations of genes controlling cytoskeletal dynamics lead to severe neurologic diseases. For example, mutations in the genes *DCX* (*DOUBLECORTIN*) and *LIS1 (LISSENCEPHALY 1)*, encoding for microtubule associated proteins, are associated with migration defects leading to type I lissencephaly, which consists in the lack of development of brain folds (gyri) and grooves (sulci) [69, 70].

In addition to defects in the development of the nervous system, alterations of the cytoskeleton can affect the maintenance of a functional neuronal network, and therefore leading to neurodegenerative disorders. In fact, several evidence indicate that tubulin acetylation is involved in neurodegenerative diseases, such as Huntington's disease (HD) and Parkinson's disease (PD) [71-73]. Moreover, the disarray of MTs and AFs represents one of the early events in the degenerative process of neurons exposed to oxidative stress [74-77]. Furthermore, MTs dysfunction has been observed in a model of Parkinson's disease where inhibition of the mitochondrial Complex I leads to accumulation of reactive oxygen species (ROS) [78, 79]. Importantly, the exact sequence of events leading to neuronal death as well as the molecular determinants for the “dying back” type of axonopathy (where progressive axonal degeneration begins distally and spreads proximally to the cell body), is still obscure and no therapy currently exists to treat the neurodegenerative progression. It has been proposed that actin-glutathionylation has a role in the pathogenesis of Freidreich's ataxia, and further studies will expand knowledge in this topic, and probably lead to an antioxidant therapeutic approach [68]. Thus, understanding the mechanisms controlling cytoskeletal rearrangements during neuronal differentiation and functionality may lead to the development of an effective therapy, which could be effective to a wide spectrum of neurodegenerative diseases.

## MUTATIONS LEADING TO NEUROLOGICAL DISEASES

Numerous studies revealed that human mutations of α- and β-tubulin genes (*TUBA1A, TUBA8, TUBB2A, TUBB4A, TUBB2B, TUBB3, TUBB*) lead to developmental brain abnormalities, i.e. lissencephaly, polymicrogyria, abnormal basal ganglia as well as cerebellar and brainstem hypoplasia [80-90]. Importantly, these mutations lead to defective cell migration and are causative of neurodevelopmental disorders (see Supplementary Table 1).

However, following the preliminary experience with the H-ABC syndrome (Hypomyelination with atrophy of the basal ganglia and cerebellum), which is related to dominant mutations of *TUBB4A* it has become evident that disorders of the cytoskeletal proteins, and tubulins in particular, are likewise responsible for neurodegenerative diseases mechanisms [90]. Recently an exome wide analysis of 363 index cases with familial ALS (FALS), revealed an excess of patient variants within *TUBA4A*, the gene encoding the Tubulin, Alpha 4A protein, further emphasizing the role of cytoskeletal defects in ALS. Functional analyses revealed that TUBA4A mutants destabilize the microtubule network, diminishing its repolymerization capability [91].

Moreover, the dissociation and the altered organization of the axonal microtubule-associated protein Tau, together with cytoskeletal disruptions are present in AD patients [92]. And, the neurodegenerative disease Troyer syndrome hereditary spastic paraplegia, caused by deficiency of spastin, which controls microtubule stability deficiency, is characterized by a disruption of both synaptic development and neuronal survival [93]. With these evidences, it is comprehensible how alterations of the cytoskeleton may cause severe abnormalities of the nervous system [94].

In addition to the above mentioned mutations in genes encoding tubulins, several evidence of the fine cytoskeletal regulation needed for the proper neuronal development come from the model of the ‘mouse with progressive motor neuronopathy’ (*pmn*), which develops a progressive caudio-cranial degeneration of the motor axons, leading to death by respiratory failure (four weeks after birth) [95]. *pmn* mice result from a spontaneous mutation in the tubulin-binding cofactor E (TBCE) gene [96, 97], which encodes one of at least five tubulin specific chaperones (TBCA-TBCE) known to promote tubulin folding and microtubule polymerization [98, 99], essential event for proper tubulin assembly and for the maintenance of microtubules in motor axons [100]. Importantly, mutations in the *TBCE* gene leads to neurodegenerative disorders.

The *pmn* mutation, resulting in homozygous Trp524Gly substitution, affects the stability of TBCE, causing axonal microtubules loss *in vivo* and a drastic reduction in tubulin levels and microtubules densities in distal axons of *pmn* spinal motor neurons [101].

Several studies have analyzed the biological mechanisms altered in the *pmn* mice and data from these studies and from TBCE-depleted motor neuron cultures showed that loss of TBCE causes Golgi vesiculation and consecutively its fragmentation [102], that is one of the earliest features of degenerating motor neurons [103, 104]. TBCE is, in fact, preeminently expressed in motor neurons as a peripheral membrane associated protein of *cis*-Golgi membranes [97], where it regulates the nucleation and the growth of Golgi-derived microtubules.

*pmn* mutation severely affects the microtubule polymerization and increase the level of soluble tubulin [105]. TBCE depleted NSC34 motor neurons treated with nocodazole, a microtubules-disrupting drug, are defective in Golgi-derived microtubules, their growth is much slower than that of control motor neurons, in which instead small microtubules begin to form immediately after nocodazole washout [101].

Loss of TBCE in *pmn* motor neurons also causes a reduction of the levels of COPI subunits (β and ε COP subunits, which form a protein complex that coats vesicles and transport proteins from the *cis* end of the Golgi complex back to the rough endoplasmic reticulum or ER, and between Golgi compartments) and an alterated recruitment of the tethering factor p115 (a COPI interactor factor) and the Golgi matrix protein GM130 at the Golgi level [101].

The transport of COPI vesicles between Golgi compartments is mediated by the tethering factor p115 that localizes at *cis*- and *medial*-Golgi [106] and, in this location, p115 has been proposed to drive the binding of COPI vesicles to the β-COP subunit [107] and to the *cis*-Golgi protein GM130 [108].

*pmn* motor neurons present decreased p115 and GM130 immunoreactivity at Golgi membranes suggesting that the loss of COPI coat affects the membrane recruitment of these tethering factors [101]. The fusion of COPI vesicles with their target membranes also requires the pairing of ER/Golgi vesicle or v-SNAREs (SNAP -Soluble NSF Attachment Protein- REceptor) with their cognate target or t-SNAREs [109].

COPI subunits degradation in *pmn* motor neurons impairs the pairing of ER/Golgi SNARE and the recycling of v-SNAREs like GS15 and GS28 [110, 111]. Therefore, it has been hypothesized that Golgi vesiculation caused by the loss of TBCE in *pmn* motor neurons is due to an impaired SNARE pairing and, in fact, the TBCE transgenic complementation of *pmn* mice restores normal p115 labeling and normalizes the pathological increase of v-SNAREs GS28-labeled Golgi elements [101].

To support the hypothesis of TBCE involvement in Golgi-derived microtubules polymerization and COPI vesicles formation, Bellouze et al. have demonstrated that the overexpression of Arf1, a small GTPase of the Ras superfamily [112], that catalyzes the fusion of the vesicle with the target membrane [113] or the constitutively active Arf1 mutant Q71L in TBCE-depleted NSC34 motor neuron cells increases recruitment of TBCE to the Golgi and strongly prevents alterations of Golgi derived microtubules, while on the other side, the inhibition of Arf1 shifts TBCE form Golgi to cytosol and also decreases the number of Golgi derived microtubules [101].

These data suggest the existence of a link between ARF1 and TBCE in tubulin polymerization/COP1 formation at the Golgi level and that the defective coordination of this cross-talk contributes to motor neuron degeneration and dysfunction. Based on these studies, it is clear that alteration of the fine control of the cytoskeletal organization lead to pathologic neuronal phenotypes and understanding the mechanism underlying the putative signaling defects is required to characterize their pathogenesis.

Another known mechanisms regulating cytoskeletal organization that has been linked to human neuronal pathology is the Rho/ROCK pathway. In fact, Rho GTPase family proteins have relevant functions in regulating various aspects of cytoskeletal neuronal development, proliferation, migration and synaptogenesis [114]. In particular, Rho/ROCK signaling is able to modulate growth cone stability by regulating actin dynamics and therefore controlling axon elongation [115, 116]. Importantly, pharmacological inhibition of ROCK is able to enhance the regeneration of the optic nerve axons after lesions and to significantly attenuate the dopaminergic cell loss in the MTP mouse model of Parkinson's disease [117].

Moreover, a form of intellectual disability is due to mutations of the OPHN1 (Oligophrenin-1) gene [118, 119], which encodes for a Rho-GTPase-activating protein promoting GTP hydrolysis of Rho subfamily members, thus controlling the contractile properties of the actin/myosin complex. In fact, the *Ophn1*^−/−^ mouse model reveals altered morphological features of the neurons and decreased synaptic vesicle endocytosis, supporting defects of the cytoskeletal organization [120]. Moreover, the relevance of the Rho pathway in controlling the cytoskeleton is confirmed by the fact that the formation of filopodia is a process mediated by the activity of cdc42, member of the Rho GTPase family [121], and the formation of lamellipodia is regulated by Rac, another member of the Rho GTPase family [122]. Therefore, the fine control of several signaling pathways necessary to modulate cellular shape and motility is central to the organization of a functional cytoskeleton and to the cellular well-being.

## IPSC TECHNOLOGY AS A MODEL TO RECAPITULATE THE REARRANGEMENTS OF THE CYTOSKELETON DURING NEUROGENESIS

**Figure 4:**
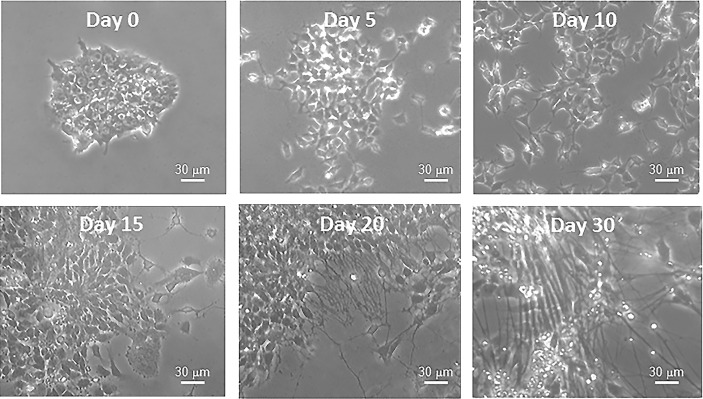
Bright field photographs of iPSCs before (Day 0) and during differentiation (Days 5, 10, 15, 20, 30) into neurons The images show the changes in cell morphology, driven by cytoskeletal rearrangements, that the cells encounter during neuronal differentiation.

At present, it has been difficult to investigate the molecular and biochemical details of the complex process of neurogenesis in animal models. These difficulties arise from the fact that it is a complex tridimensional and non-synchronous event, nearly impossible to analyze during human development without interfering with the integrity of the developing embryo. Importantly, the development of the induced pluripotent stem cell (iPSC) technology allows to solve the inaccessibility problem linked to the study of neurogenesis. In fact, iPSCs, which are stem cells reprogrammed from adult somatic cells of different embryonic origin (endoderm, ectoderm and mesoderm), can be differentiated in a dish into functional mature neurons (Figure 4) [123]. iPSCs can be easily used to investigate the details of specific molecular mechanisms and morphological changes occurring during different phases of *in vitro* differentiation [124]. This process, named *in vitro* neurogenesis, can be investigated in any laboratory and many studies are currently advancing to understand the molecular and biochemical underpinnings of human neurogenesis, particularly those associated to poorly known human pathologies. For example, the length of the neurites, the number of branches departing from one neurite and the branching levels are parameters used to measure the maturity level of *in vitro* neuronal cultures. In detail, primary neurites are the processes projecting directly from the cell body (branching level 0), secondary neurites are those processes that branch from any primary neurite (branching level 1) and tertiary processes are those that projected from any secondary neurite (branching level 2). Thus, these features allow, not only to compare the maturity level of different neuronal cell cultures from different genetic backgrounds or following different pharmacological treatments, but they also allow to thoroughly investigate the processes modulating neurite formation and outgrowth. We expect that advances in fluorescent labeling, super-resolution fluorescence microscopy, and electron microscopy will greatly accelerate this research. In addition, a precise dissection of the molecular mechanisms of cytoskeletal crosstalk will also require complementary *in vitro* experiments.

A systematic and integrated analysis will help us to understand MT, AF and IF functions and dynamics in the nervous system during development and disease. One possibility to deeply investigate the mechanisms of cytoskeletal rearrangements during neurogenesis is offered by iPSCs. In fact, iPSCs can be differentiated *in vitro* into several cell types, including neurons and glial cells, allowing to follow their molecular, biochemical and morphological features during human neurogenesis/neuritogenesis in a dish. iPSCs have been compared to ESCs and many studies demonstrated that they present molecular and cellular properties very similar to ESCs [125, 126]. Unfortunately, very little is known about the cytoskeleton of human ESCs and even less about the cytoskeleton of iPSCs. Therefore, it is necessary to further the investigation of the cytoskeletal arrangements before and during differentiation of iPSCs into different cell fates and it is also important to understand the molecular determinants that regulate and are regulated by cytoskeletal rearrangements. One recent line of evidence suggests that matrix elasticity/stiffness regulates the protein levels of the nucleoskeletal lamin A, of actin-myosin expression, and most interestingly, the matrix stiffness modulates cell fate determination [127-129]. These studies suggest that the rearrangement of the cytoskeleton is not only orchestrated by the nuclear transcriptional activity, but that it plays an active role in the determination of different cell lineage commitment. In fact, these evidences suggest that the extra-cellular environment is able to communicate with the intra-cellular cytoskeleton and with the transcriptional machinery in the nucleus, thus being directly responsible for cellular differentiation and cell fate commitment. Importantly, the nucleoskeleton changes during differentiation, but a deep understanding of nuclear lamina changes during cell differentiation is still preliminary. A seminal study to understand the relevance of lamins in stem cell and differentiation has been performed by Swift et al. [129]. They demonstrated that Lamin A levels have a role in mechanosensitive differentiation. Thus, it is tempting to hypothesize that the differential expression of specific lamins may regulate the tissue- specific gene expression, and that tissue mechanics may account for tissue-specific gene expression.

In addition to the iPSC technology, the recently developed genome editing technique known as CRISPR/Cas9 offers great advantages to cellular reprogramming, and a great advancement to the reliability of iPSC model for human diseases and for its applications in translational medicine. The CRISPR/Cas9 technology for editing genomes, allows scientists to make changes in DNA. In particular, it offers the possibility to insert or remove genomic mutation in specific genes and to correct known mutations. Thus, the CRISPR/Cas9 technology allows to obtain isogenic control iPSCs, where the mutations present in the patients are reversed to the wild type sequence. Importantly, this technique allows to perform studies on iPSCs obtained from specific patients and on genetically corrected cells that have the same genetic background of the affected cells [130-132]. Moreover, among the possible applications of the CRISPR/Cas9 there is the possibility to perform temporal control of gene expression or deletion, by combining it with the Flp/FRT and Cre/LoxP system, which have been already used to obtain inducible gene knockout in iPSCs [133].

Alternative techniques for studying the generation and differentiation of neurons in their *in vivo* environment account on *in utero* electroporation, which allows transfection of plasmid DNA into restricted areas of the brain and is performed on small embryos through electroporation. This helps to study *in vivo* processes like cell differentiation, cell migration and axon guidance [22, 134, 135], but it also presents disadvantages. In fact, it is a model that, even if it is performed with high technical precision, it needs to take into account the small size of the murine embryos and the possibility to target non specific cells (*i.e.* the mesenchymal cells) and therefore, the effects observed may result from the influence of different cell types on the developing neural tube. In addition to this, it can not be performed early in development (*i.e.* during gastrulation or at the neural plate stage) and this impedes the possibility that the gene of interest may have an effect very early in development. Moreover, it is limited to investigations in the murine/rodent models and it is possibile that it is not suitable to model several human neurologic disorders. For example, therapeutic approaches developed in Amyotrophic lateral sclerosis (ALS) animals with encouraging results were not successful in human clinical trials [136].

In conclusion, a new methodology that allows to study neurogenesis in a 3D system is the cerebral organoid technology, that has been obtained from human iPSCs and allows to develop mini-brains *in vitro*, which resembles features of human cortical development and it has been used to model microcephaly due to *CDK5RAP2* mutation [137]. Thus, this *in vitro* culture system has the great potential to model still poorly known human neurodevelopmental and neurological pathologies.

An example on how several technologies can be integrated to establish a reliable human disease model based on the iPSC technology is offered by Chen et al. [138]. In fact, Chen et al. used iPSCs to obtain enriched and synchronized motor neurons and non-motor neuron cultures from ALS patients and isogenic control (by using the TALEN gene editing technique). These study allowed to unveil the presence of neurofilaments inclusions as an early event in MN neurite degeneration and pave the way to the possibility to target neurofilament control for clinical applications [138]. At present the limitations of the iPSC system consist mainly in the impossibility to control tightly neuronal cell body clustering and the criss-crossing of axons with those from other neuronal types, but some strategies can be used to overcome these difficulties. For example, Taylor et al. [139] described the method of a microfluidic culture platform (or compartmentalized ‘Campenot’ chamber) able to probe axons independently from cell bodies, thus facilitating studies dealing with axonal biology. In addition to this, a recent study by the Haase laboratory [140] showed how to isolate 100% pure human iPSC-derived motor neurons by a FACS double selection based method, thus improving iPSC-based disease modeling and drug testing in motor neuron disorders.

## CONCLUSIONS

In light of the studies related to the discovery of human mutations leading to neurologic diseases and the new evidence on the cytoskeleton as an active player of cell fate determination, it is compulsory to deeply investigate the mechanisms regulating and regulated by the cytoskeleton. This may offer the opportunity to understand the physio-pathology of many human neurologic diseases and to pave the way for future therapeutic intervention.

Moreover, recent advances in live imaging will allow to reveal the dynamics of cytoskeletal organization during neural development and the combination of these methodologies together with the iPSC technology will speed the pace for therapeutic intervention of many neurologic diseases [as reviewed in 141].

## SUPPLEMENTARY MATERIAL TABLE



## Abbreviations

AF: actin filaments
IF: intermediate filaments
AD: Alzheimer's disease
ALS: Amyotrophic lateral sclerosis
ER: endoplasmic reticulum
ESC: embryonic stem cell
HD: Huntington's disease
iPSC: induced pluripotent stem cells
MT: microtubules
PD: Parkinson's disease
ROS: reactive oxygen species.
