# Supplementary material for: The cytoskeletal arrangements necessary to neurogenesis
Source: Oncotarget. 2016 Jan 7;7(15):19414–29. doi: 10.18632/oncotarget.6838 (PMC4991392; doi:10.18632/oncotarget.6838)
Supplement: Supplementary file 1 [file oncotarget-07-19414-s001.pdf]

The cytoskeletal arrangements necessary to neurogenesis

Supplementary Material

Supplementary Table 1.

TABLE SUMMARIZING THE DIFFERENT TUBULIN AND TUBULIN CHAPERONE MUTATIONS WITH THE ASSOCIATED DISEASE PHENOTYPE AND CORRESPONDING REFERENCES.

Gene	*cDNA (Mutations)*	*Protein*	*Exon*	*Phenotype*	References
**TUBA1A**	c.163G>A	p.E55K	2	Lissencephaly	Keays *et al.*, 2007,<br>Poirier et al., 2007<br>Bahi-Buisson *et al.*, 2008<br>Morris-Rosendahl *et al.*, 2008<br>Kumar et al., 2010<br>Jansen *et al.*, 2011
	c.410T>A	p.V137D	4		
	c.562A>C	p.I188L	4		
	c.629A>G	p.Y210C	4		
	c.652G>T	p.D218Y	4		
	c.808G>A	p.A270T	4		
	c.986A>G	p.N329S	4		
	c.1204C>T	p.R402C	4		
	c.1205G>A	p.R402H	4		
	c.1205G>T	p.R402L	4		
	c.1256C>T	p.S419L	4		
	c.1265G>A	p.R422H	4		
	c.1274T>A	p.M425K	4		
	c. 1129A>G	p.M377V	4		
**TUBA1A**	c.167C>T	p.T56M	2	Lissencephaly (in fetus)	Fallet-Bianco *et al.*, 2008<br>Kumar et al., 2010<br>Lecourtois *et al.*, 2010<br>Bahi-Buisson *et al.*, 2014
	c.214C>T	p.P72S	2		
	c.274C>G	p.L92V	3		
	c.302A>G	p.N101S	4		
	c.712A>G	p.I1238V	4		
	c.787C>A	p.P263T	4		
	c.790C>T	p.R264H	4		
	c.856C>T	p.L286F	4		
	c.908T>G	p.V303G	4		
	c.959G>A	p.R320H	4		

	c.978A>C	p.K326N	4		
	c.1112T>A	p.V371E	4		
	c.1204C>T	p.R402C	4		
	c.1205G>A	p.R402H	4		
	c.1226T>C	p.V409A	4		
	c.1265G>A	p.R422H	4		
	c.1285G>C	p.E429Q	4		
**TUBA1A**	c.1096G>A	p.G366R	4	Lissencephaly, agenesis of corpus callosum and cerebellar hypoplasia	Okumura *et al.*, 2012
**TUBA1A**	c.337G>A	p.E113K	4	Pachygyria	Poirier et al., 2007
	c.790C>T	p.R264C	4		Bahi-Buisson *et al.*, 2008
	c.1105G>A	p.A369T	4		Bahi-Buisson *et al.*, 2014
	c.1190T>C	p.L397P	4		
	c.1225G>A	p.V409I	4		
	c.1264C>T	p.R422C	4		
	c.1306G>T	p.G436R	4		
**TUBA1A**	c.13A>C	p.I5L	2	Polymicrogyria	Jansen et al., 2011
	c.367C>T	p.R123C	4		Poirier *et al.*, 2013
	c.481T>C	p.Y161H	4		Bahi-Buisson *et al.*, 2014
	c.703G>T	p.V235L	4		
	c.1168C>T	p.R390C	4		
	c.1186G>T	p.D396Y	4		
**TUBA1A**	c.473C>T	p.S158L	4	Polymicrogyria (in fetus)	Bahi-Buisson *et al.*, 2014
	c.641G>A	p.R214H	4		
**TUBA1A**	c.1169G>A	p.R390H	4	Poyimicrogyria and mid-hindbrain dysgenesis	Zanni *et al.*, 2013
**TUBA1A**	c.79G>C	p.E27Q	2	Malformation of cortical development	Shimojima *et al.*, 2014
**TUBA1A**	c.808G>T	p.A270S	4	Eyes abnormalities and brain malformation	Myers *et al.*, 2015
**TUBA1A**	c.641G>A	p.R214H	4	Cerebellar dysplasia	Oegema et al.,2015
	c.655A>G	p.I219V	4		

**TUBA1A**	c.190C>T	p.R64W	2	Cortical dysgenesis	Yokoi *et al*., 2015
	c.74G>T	p.C25F	2		
**TUBB2A**	c.743C>T	p.N247L	4	Infantile-onset epilepsy	Cushion *et al*., 2014
	c.741C>G	p.A248V	4		
**TUBB2A**	c.302G>A	p.G98R	4	Lissencephaly (in fetus)	Bahi-Buisson *et al*., 2014
	c.716G>T	p.C239F	4		
	c.745G>C	p.D249H	4		
**TUBB2B**	c.511C>A	p.P171T	4	Polymicrogyria	Jaglin *et al*., 2009<br>Bahi-Buisson *et al*., 2014
	c.514T>C	p.S172P	4		
	c.605T>C	p.I202T	4		
	c.629T>C	p.I210T	4		
	c.683T>C	p.L288P	4		
	c.793T>C	p.F265L	4		
	c.935C>T	p.T312M	4		
	c.1106G>T	p.G369V	4		
**TUBB2B**	c.514T>C	p.S172P	4	Polymicrogyria (in fetus)	Jaglin *et al*., 2009<br>Bahi-Buisson *et al*., 2014
	c.518C>T	p.P173L	4		
	c.742G>A	p.A248T	4		
**TUBB2B**	c.1261G>A	p.E421K	4	Polymicrogyria, congenital fibrosis of the extra ocular muscles and axon dysinnervation	Cederquist *et al*., 2012
**TUBB2B**	c.419G>C	p.G140A	4	Malformation of cortical development and deficits in axonal guidance	Romaniello *et al*., 2012
**TUBB2B**	c.743C>T	p.A248V	4	Polymicrogyria with dysmorphic basal ganglia	Amrom *et al*., 2014
	c.1139G>T	p.R380L	4		
**TUBB2B**	c.38G>C	p.G13A	1	Cerebellar dysplasia	Oegema et al.,2015
**TUBB3**	c.1162A>G	p.M388V	4	Lissencephaly (in fetus)	Poirier *et al*., 2010
**TUBB3**	c.784C>T	p.R262C	4	Commissural axon, basal ganglia malformation	Tischfield *et al*., 2010
	c.904G>A	p.A302T	4		
	c.1138C>T	p.R380C	4		
	c.1249G>A	p.D417N	4		

**TUBB3**	c.785G>A	p.R262H	4	Commissural axon, basal ganglia malformation, congenital oculomotor nerve hypoplasia and peripheral axon degeneration	Tischfield *et al.*, 2010
	c.1228G>A	p.E410K	4		
**TUBB3**	c.244G>A	p.G82R	3	Cortical disorganization, axonal abnormalities associated with pontocerebellar hypoplasia	Poirier *et al.*, 2010
	c.633G>A	p.E205K	4		
	c.905C>T	p.A302V	4		
	c.967A>G	p.M323V	4		
	c.1162A>G	p.M388V	4		
**TUBB3**	c.862G>A	p.Glu288Lys	4	Cerebellar dysplasia	Oegema et al.,2015
	c.1070C>T	p.P357L	4		
**TUBB4A**	c.745G>A	p.D249N	4	Leukodystrophy hypomyelination with atrophy of the basal ganglia and cerebellum	Simons et al.,2013<br>Carvalho et al., 2014<br>Ferreira et al., 2014<br>Hamilton et al., 2014<br>Miyatake et al., 2014<br>Tonduti *et al.,* 2015
	c.4C>T	p.R2W	1		
	c.5G>A	p.R2G	1		
	c.533C>G	p.T178R	4		
	c.533C>T	p.T178M	4		
	c.544C>A	p.P182T	4		
	c.716G>T	p.C239F	4		
	c.730G>A	p.G244S	4		
	c.731G>T	p.G244V	4		
	c.731G>A	p.G244D	4		
	c.785G>A	p.R262H	4		
	c.968T>G	p.M323R	4		
	c.1054G>A	p.A352T	4		
	c.1061G>A	p.C354Y	4		
	c.1099T>A	p.F367I	4		
	c.1099T>C	p.F367L	4		
	c.1162A>G	p.M388V	4		
	c.1163T>C	p.M388T	4		
	c.1164G>A	p.M388I	4		
	c.1181T>C	p.F294C	4		
	c.1228G>A	p.E410K	4		

**TUBA4A**	c.958C>T	p.R320C	4	Familial amyotrophic lateral sclerosis	Smith *et al*., 2014
	c.959G>A	p.R320H	4		
	c.1147C>A	p.A383T	4		
	c.1220G>A	p.W407X	4		
**TUBB4A**	c.467G>T	p.R156L	4	Isolated hypomyelinating leukodystrophy	Pizzino et al., 2014<br>Purnell *et al*., 2014
	c.763G>A	p.V255I	4		
	c.845G>C	p.R282P	4		
	c.874C>A	p.Q292K	4		
	c.1172G>A	p.R391H	4		
**TUBB4A**	c.4C>G	p.R2G	4	Whispering dysphonia	Hersheson et al., 2013<br>Lohmann et al., 2013
**TUBB4A**	c.900G>T	p.M300I	4	H-ABC and DYT4	Erro et al., 2015
	c.941C>T	p.A314V	4		
**TUBA8**	c.76-10610 del(gttgcttccctctc)	p.del 2-75	Intron1	Polymicrogyria	Abdollahi *et al*., 2009
**TUBB5**	c.895A>G	p.M299V	4	Microcephaly with structural brain abnormalities	Breuss *et al*., 2012
	c.1057G>A	p.V353I	4		
	c.1201G>A	p.E401K	4		
**TBCE**	c.155-166del 12	p.del 52-55	2	Hypoparathyroidism-retardation-dysmorphism syndrome and Kenny Caffey syndrome	Parvari *et al*., 2002<br>Padidela *et al*., 2009
**TBCE**	c.66delAG,<br>c.1113T>A	p.V23fs48X,<br>p.C371X	1,<br>12	Hypoparathyroidism-retardation-dysmorphism syndrome	Parvari *et al*., 2002

Glossary: H-ABC: hypomyelination with atrophy of the basal ganglia and cerebellum (see van der Knaap et al., 2002); **DYT4**: dystonia-4, (or whispering dysphonia), progressive laryngeal dysphonia followed by the involvement of other muscles, such as the neck or limbs, it may be accompanied by an ataxic gait (see Hersheson et al., 2013 and Lohman et al., 2013).

References of Supplementary Table 1.

- Abdollahi MR, Morrison E, Sirey T, Molnar Z, Hayward BE, Carr IM, Springell K, Woods CG, Ahmed M, Hattingh L, Corry P, Pilz DT, Stoodley N, Crow Y, Taylor GR, Bonthron DT, Sheridan E. Mutation of the variant alpha-tubulin TUBA8 results in polymicrogyria with optic nerve hypoplasia. Am J Hum Genet. 2009; 85:737-44.
- Amrom D, Tanyalçin I, Verhelst H, Deconinck N, Brouhard GJ, Décarie JC, Vanderhasselt T, Das S, Hamdan FF, Lissens W, Michaud JL, Jansen AC. Polymicrogyria with dysmorphic basal ganglia? Think tubulin! Clin Genet. 2014; 85:178-83.
- Bahi-Buisson N, Poirier K, Boddaert N, Saillour Y, Castelnau L, Philip N, Buyse G, Villard L, Joriot S, Marret S, Bourgeois M, Van Esch H, Lagae L, Amiel J,Hertz-Pannier L, Roubertie A, Rivier F, Pinard JM, Beldjord C, Chelly J. Refinement of cortical dysgeneses spectrum associated with TUBA1A mutations. J Med Genet. 2008; 45:647-53.
- Bahi-Buisson N, Poirier K, Fourniol F, Saillour Y, Valence S, Lebrun N, Hully M, Bianco CF, Boddaert N, Elie C, Lascelles K, Souville I; LIS-Tubulinopathies Consortium, Beldjord C, Chelly J. The wide spectrum of tubulinopathies: what are the key features for the diagnosis? Brain. 2014; 137:1676-700.
- Breuss M, Heng JI, Poirier K, Tian G, Jaglin XH, Qu Z, Braun A, Gstrein T, Ngo L, Haas M, Bahi-Buisson N, Moutard ML, Passemard S, Verloes A, Gressens P, Xie Y, Robson KJ, Rani DS, Thangaraj K, Clausen T, Chelly J, Cowan NJ, Keays DA. Mutations in the β-tubulin gene TUBB5 cause microcephaly with structural brain abnormalities. Cell Rep. 2012; 2:1554-62.
- Carvalho D, Santos S, Martins B, Marques FP. TUBB4A novel mutation reinforces the genotype-phenotype correlation of hypomyelination with atrophy of the basal ganglia and cerebellum. Brain. 2015; 138:e327.
- Cederquist GY, Luchniak A, Tischfield MA, Peeva M, Song Y, Menezes MP, Chan WM, Andrews C, Chew S, Jamieson RV, Gomes L, Flaherty M, Grant PE, Gupta ML Jr, Engle EC. An inherited TUBB2B mutation alters a kinesin-binding site and causes polymicrogyria, CFEOM and axon dysinnervation. Hum Mol Genet. 2012; 21:5484-99.
- Cushion TD, Paciorkowski AR, Pilz DT, Mullins JG, Seltzer LE, Marion RW, Tuttle E, Ghoneim D, Christian SL, Chung SK, Rees MI, Dobyns WB., Seo-Kyung Chung,Mark I. Rees, William B. Dobyns De Novo Mutations in the Beta-Tubulin Gene *TUBB2A* Cause Simplified Gyral Patterning and Infantile-Onset Epilepsy. Am J Hum Genet. 2014; 94: 634–641.
- Erro R, Hersheson J, Ganos C, Mencacci NE, Stamelou M, Batla A, Thust SC, Bras JM, Guerreiro RJ, Hardy J, Quinn NP, Houlden H, Bhatia KP. H-ABC syndrome and DYT4: Variable expressivity or pleiotropy of TUBB4 mutations? Mov Disord. 2015; 30:828-33.
- Fallet-Bianco C, Loeuillet L, Poirier K, Loget P, Chapon F, Pasquier L, Saillour Y, Beldjord C, Chelly J, Francis F. Neuropathological phenotype of a distinct form of lissencephaly associated with mutations in TUBA1A. Brain. 2008; 131:2304-20.
- Ferreira C, Poretti A, Cohen J, Hamosh A, Naidu S. Novel TUBB4A mutations and expansion of the neuroimaging phenotype of hypomyelination with atrophy of the basal ganglia and cerebellum (H-ABC). Am J Med Genet A. 2014; 164A:1802-7.
- Hamilton EM, Polder E, Vanderver A, et al. Hypomyelination with atrophy of the basal ganglia and cerebellum: further delineation of the phenotype and genotype-phenotype correlation. Brain. 2014; 137(Pt 7):1921-30.

- Hersheson J, Mencacci NE, Davis M, et al. Mutations in the autoregulatory domain of beta-tubulin 4a cause hereditary dystonia. Ann Neurol. 2013; 73:546–53.
- Hersheson J, Mencacci NE, Davis M, MacDonald N, Trabzuni D, Ryten M, Pittman A, Paudel R, Kara E, Fawcett K, Plagnol V, Bhatia KP, Medlar AJ, Stanescu HC, Hardy J, Kleta R, Wood NW, Houlden H. Mutations in the autoregulatory domain of β-tubulin 4a cause hereditary dystonia. Ann Neurol. 2013; 73:546-53.
- Jaglin XH, Poirier K, Saillour Y, Buhler E, Tian G, Bahi-Buisson N, Fallet-Bianco C, Phan-Dinh-Tuy F, Kong XP, Bomont P, Castelnau-Ptakhine L, Odent S, Loget P, Kossorotoff M, Snoeck I, Plessis G, Parent P, Beldjord C, Cardoso C, Represa A, Flint J, Keays DA, Cowan NJ, Chelly J. Mutations in the beta-tubulin gene TUBB2B result in asymmetrical polymicrogyria. Nat Genet. 2009; 41:746-52.
- Jansen AC, Oostra A, Desprechins B, De Vlaeminck Y, Verhelst H, Régal L, Verloo P, Bockaert N, Keymolen K, Seneca S, De Meirleir L, Lissens W. TUBA1A mutations: from isolated lissencephaly to familial polymicrogyria. Neurology. 2011; 76:988-92.
- Keays DA, Tian G, Poirier K, Huang GJ, Siebold C, Cleak J, Oliver PL, Fray M, Harvey RJ, Molnár Z, Piñon MC, Dear N, Valdar W, Brown SD, Davies KE, Rawlins JN, Cowan NJ, Nolan P, Chelly J, Flint J. Mutations in alpha-tubulin cause abnormal neuronal migration in mice and lissencephaly in humans.Cell. 2007; 128:45-57.
- Kumar RA, Pilz DT, Babatz TD, Cushion TD, Harvey K, Topf M, Yates L, Robb S, Uyanik G, Mancini GM, Rees MI, Harvey RJ, Dobyns WB. TUBA1A mutations cause wide spectrum lissencephaly (smooth brain) and suggest that multiple neuronal migration pathways converge on alpha tubulins. Hum Mol Genet. 2010; 19:2817-27.
- Lecourtois M, Poirier K, Friocourt G, Jaglin X, Goldenberg A, Saugier-Veber P, Chelly J, Laquerrière A. Human lissencephaly with cerebellar hypoplasia due to mutations in TUBA1A: expansion of the foetal neuropathological phenotype. Acta Neuropathol. 2010 Jun;119:779-89.
- Lohmann K, Wilcox RA, Winkler S, Ramirez A, Rakovic A, Park JS, Arns B, Lohnau T, Groen J, Kasten M, Brüggemann N, Hagenah J, Schmidt A, Kaiser FJ,Kumar KR, Zschiedrich K, Alvarez-Fischer D, Altenmüller E, Ferbert A, Lang AE, Münchau A, Kostic V, Simonyan K, Agzarian M, Ozelius LJ, Langeveld AP, Sue CM, Tijssen MA, Klein C. Whispering dysphonia (DYT4 dystonia) is caused by a mutation in the TUBB4 gene. Ann Neurol. 2013; 73:537-45.
- Miyatake S, Osaka H, Shiina M, Sasaki M, Takanashi J, Haginoya K, Wada T, Morimoto M, Ando N, Ikuta Y, Nakashima M, Tsurusaki Y, Miyake N,Ogata K, Matsumoto N, Saitsu H. Expanding the phenotypic spectrum of TUBB4A-associated hypomyelinating leukoencephalopathies. Neurology. 2014; 82:2230-7.
- Morris-Rosendahl DJ, Najm J, Lachmeijer AM, Sztriha L, Martins M, Kuechler A, Haug V, Zeschnigk C, Martin P, Santos M, Vasconcelos C, Omran H, Kraus U, Van der Knaap MS, Schuierer G, Kutsche K, Uyanik G. Refining the phenotype of alpha-1a Tubulin (TUBA1A) mutation in patients with classical lissencephaly. Clin Genet. 2008; 74:425-33.
- Myers KA, Bello-Espinosa LE, Kherani A, Wei XC, Innes AM. TUBA1A Mutation Associated With Eye Abnormalities in Addition to Brain Malformation. Pediatr Neurol. 2015; 53:442-4.
- Oegema R, Cushion TD, Phelps IG, Chung SK, Dempsey JC, Collins S, Mullins JG, Dudding T, Gill H, Green AJ, Dobyns WB, Ishak GE, Rees MI, Doherty D. Recognizable cerebellar dysplasia associated with mutations in multiple tubulin genes. Hum Mol Genet. 2015; 24:5313-25.

- Okumura A, Hayashi M, Tsurui H, Yamakawa Y, Abe S, Kudo T, Suzuki R, Shimizu T, Shimojima K, Yamamoto T. Lissencephaly with marked ventricular dilation, agenesis of corpus callosum, and cerebellar hypoplasia caused by TUBA1A mutation. Brain Dev. 2013; 35:274-9.
- Padidela R, Kelberman D, Press M, Al-Khawari M, Hindmarsh PC, Dattani MT. Mutation in the TBCE gene is associated with hypoparathyroidism-retardation-dysmorphism syndrome featuring pituitary hormone deficiencies and hypoplasia of the anterior pituitary and the corpus callosum. J Clin Endocrinol Metab. 2009; 94:2686-91.
- Parvari R, Hershkovitz E, Grossman N, Gorodischer R, Loeys B, Zecic A, Mortier G, Gregory S, Sharony R, Kambouris M, Sakati N, Meyer BF, Al Aqeel AI, Al Humaidan AK, Al Zanhrani F, Al Swaid A, Al Othman J, Diaz GA, Weiner R, Khan KT, Gordon R, Gelb BD; HRD/Autosomal Recessive Kenny-Caffey Syndrome Consortium. Mutation of TBCE causes hypoparathyroidism-retardation-dysmorphism and autosomal recessive Kenny-Caffey syndrome. Nat Genet. 2002; 32:448-52.
- Pizzino A, Pierson TM, Guo Y, Helman G, Fortini S, Guerrero K, Saitta S, Murphy JL, Padiath Q, Xie Y, Hakonarson H, Xu X, Funari T, Fox M, Taft RJ, van der Knaap MS, Bernard G, Schiffmann R, Simons C, Vanderver A. TUBB4A de novo mutations cause isolated hypomyelination. Neurology.2014; 83:898-902.
- Poirier K, Keays DA, Francis F, Saillour Y, Bahi N, Manouvrier S, Fallet-Bianco C, Pasquier L, Toutain A, Tuy FP, Bienvenu T, Joriot S, Odent S, Ville D, Desguerre I, Goldenberg A, Moutard ML, Fryns JP, van Esch H, Harvey RJ, Siebold C, Flint J, Beldjord C, Chelly J. Large spectrum of lissencephaly and pachygyria phenotypes resulting from de novo missense mutations in tubulin alpha 1A (TUBA1A). Hum Mutat. 2007; 28:1055-64.
- Poirier K, Saillour Y, Bahi-Buisson N, Jaglin XH, Fallet-Bianco C, Nabbout R, Castelnau-Ptakhine L, Roubertie A, Attie-Bitach T,Desguerre I, Genevieve D, Barnerias C, Keren B, Lebrun N, Boddaert N, Encha-Razavi F, Chelly J. Mutations in the neuronal ß-tubulin subunit TUBB3 result in malformation of cortical development and neuronal migration defects. Hum Mol Genet. 2010; 19:4462-73.
- Poirier K, Saillour Y, Fourniol F, Francis F, Souville I, Valence S, Desguerre I, Marie Lepage J, Boddaert N, Line Jacquemont M, Beldjord C, Chelly J, Bahi-Buisson N. Expanding the spectrum of TUBA1A-related cortical dysgenesis to Polymicrogyria. Eur J Hum Genet. 2013; 21:381-5.
- Purnell SM, Bleyl SB, Bonkowsky JL. Clinical exome sequencing identifies a novel TUBB4A mutation in a child with static hypomyelinating leukodystrophy. Pediatr Neurol. 2014; 50:608-11.
- Romaniello R, Tonelli A, Arrigoni F, Baschirotto C, Triulzi F, Bresolin N, Bassi MT, Borgatti R. A novel mutation in the β-tubulin gene TUBB2B associated with complex malformation of cortical development and deficits in axonal guidance. Dev Med Child Neurol. 2012; 54:765-9.
- Shimojima K, Narita A, Maegaki Y, Saito A, Furukawa T, Yamamoto T. Whole-exome sequencing identifies a de novo TUBA1A mutation in a patient with sporadic malformations of cortical development: a case report. BMC Res Notes. 2014; 7:465.
- Simons C, Wolf NI, McNeil N, Caldovic L, Devaney JM, Takanohashi A, Crawford J, Ru K, Grimmond SM, Miller D, Tonduti D, Schmidt JL, Chudnow RS, van Coster R, Lagae L, Kisler J, Sperner J van der Knaap MS, Schiffmann R, Taft RJ, Vanderver A. A de novo mutation in the β-tubulin gene TUBB4A results in the leukoencephalopathy hypomyelination with atrophy of the basal ganglia and cerebellum. Am J Hum Genet. 2013; 92:767-73.

- Smith BN, Ticozzi N, Fallini C, Gkazi AS, Topp S, Kenna KP, Scotter EL, Kost J, Keagle P, Miller JW, Calini D, Vance C, Danielson EW, Troakes C, Tiloca C, Al-Sarraj S, Lewis EA, King A, Colombrita C, Pensato V, Castellotti B, de Belleroche J, Baas F, ten Asbroek AL, Sapp PC, McKenna-Yasek D, McLaughlin RL, Polak M, Asress S, Esteban-Pérez J, Muñoz-Blanco JL, Simpson M; SLAGEN Consortium, van Rheenen W, Diekstra FP, Lauria G, Duga S, Corti S, Cereda C, Corrado L, Sorarù G, Morrison KE, Williams KL, Nicholson GA, Blair IP, Dion PA, Leblond CS, Rouleau GA, Hardiman O, Veldink JH, van den Berg LH, Al-Chalabi A, Pall H, Shaw PJ, Turner MR, Talbot K, Taroni F, García-Redondo A, Wu Z, Glass JD, Gellera C, Ratti A, Brown RH Jr, Silani V, Shaw CE, Landers JE. Exome-wide rare variant analysis identifies TUBA4A mutations associated with familial ALS. Neuron. 2014; 84:324-31.
- Tischfield MA, Baris HN, Wu C, Rudolph G, Van Maldergem L, He W, Chan WM, Andrews C, Demer JL, Robertson RL, Mackey DA, Ruddle JB, Bird TD, Gottlob I, Pieh C, Traboulsi EI, Pomeroy SL, Hunter DG, Soul JS, Newlin A, Sabol LJ, Doherty EJ, de Uzcátegui CE, de Uzcátegui N, Collins ML, Sener EC, Wabbels B, Hellebrand H, Meitinger T, de Berardinis T, Magli A, Schiavi C, Pastore-Trossello M, Koc F, Wong AM, Levin AV, Geraghty MT, Descartes M, Flaherty M, Jamieson RV, Møller HU, Meuthen I, Callen DF, Kerwin J, Lindsay S, Meindl A, Gupta ML Jr, Pellman D, Engle EC. Human TUBB3 mutations perturb microtubule dynamics, kinesin interactions, and axon guidance. Cell. 2010; 140:74-87.
- Tonduti D, Aiello C, Renaldo F, Dorboz I, Saaman S, Rodriguez D, Fettah H, Elmaleh M, Biancheri R, Barresi S, Boccone L, Orcesi S, Pichiecchio A, Zangaglia R, Maurey H, Rossi A, Boespflug-Tanguy O, Bertini E. TUBB4A-related hypomyelinating leukodystrophy: New insights from a series of 12 patients. Eur J Paediatr Neurol. 2015; pii: S1090-3798(15)00189-0.
- van der Knaap MS, Naidu S, Pouwels PJ, et al. New syndrome characterized by hypomyelination with atrophy of the basal ganglia and cerebellum. AJNR Am J Neuroradiol. 2002; 23(9):1466-74.
- Yokoi S, Ishihara N, Miya F, Tsutsumi M, Yanagihara I, Fujita N, Yamamoto H, Kato M, Okamoto N, Tsunoda T, Yamasaki M, Kanemura Y, Kosaki K, Kojima S, Saitoh S, Kurahashi H, Natsume J. TUBA1A mutation can cause a hydranencephaly-like severe form of cortical dysgenesis. Sci Rep. 2015; 5:15165.
- Zanni G, Colafati GS, Barresi S, Randisi F, Talamanca LF, Genovese E, Bellacchio E, Bartuli A, Bernardi B, Bertini E. Description of a novel TUBA1A mutation in Arg-390 associated with asymmetrical polymicrogyria and mid-hindbrain dysgenesis. Eur J Paediatr Neurol. 2013; 17:361-5.
